# Quality of neonatal healthcare in Kilimanjaro region, northeast Tanzania: learning from mothers' experiences

**DOI:** 10.1186/1471-2431-13-68

**Published:** 2013-05-03

**Authors:** Bernard Mbwele, Nicole L Ide, Elizabeth Reddy, Sarah A P Ward, Joshua A Melnick, Flavian A Masokoto, Rachael Manongi

**Affiliations:** 1 Kilimanjaro Clinical Research Institute, Kilimanjaro Christian Medical Center, P.O Box 2236, KCMC, Moshi, Tanzania; 2 Seattle Pacific University, 3307 3rd Ave West, Seattle, WA 98119-1997, USA; 3 Department of Medicine Duke University, P.O Box 3010, Moshi, Tanzania; 4 Division of Infectious Disease, P.O Box 3010, Moshi, Tanzania; 5 Kilimanjaro Christian Medical Centre-Duke University Collaboration, P.O Box 3010, Moshi, Tanzania; 6 Bowdoin College, Bowdoin College, 5000 South Street, Brunswick, ME 04011, USA; 7 University of Georgia, Athens, GA 39602, Greece; 8 Uru Mawela Parish, Moshi Diocese at P.O Box 3011, Moshi, Kilimanjaro, Tanzania; 9 Kilimanjaro Christian Medical University College, P.O Box 2240, KCMC, Moshi, Tanzania

**Keywords:** Neonatal, Mothers, Quality of care, Parents, Family, Satisfaction, Challenges, Kilimanjaro, Tanzania

## Abstract

**Background:**

With a decline of infant mortality rates, neonatal mortality rates are striking high in development countries particularly sub Saharan Africa. The toolkit for high quality neonatal services describes the principle of patient satisfaction, which we translate as mother’s involvement in neonatal care and so better outcomes. The aim of the study was to assess mothers’ experiences, perception and satisfaction of neonatal care in the hospitals of Kilimanjaro region of Tanzania.

**Methods:**

A cross sectional study using qualitative and quantitative approaches in 112 semi structured interviews from 14 health facilities. Open ended questions for detection of illness, care given to the baby and time spent by the health worker for care and treatment were studied. Probing of the responses was used to extract and describe findings by a mix of in-depth interview skills. Closed ended questions for the quantitative variables were used to quantify findings for statistical use. Narratives from open ended questions were coded by colours in excel sheet and themes were manually counted.

**Results:**

80 mothers were interviewed from 13 peripheral facilities and 32 mothers were interviewed at a zonal referral hospital of Kilimanjaro region. 59 mothers (73.8%) in the peripheral hospitals of the region noted neonatal problems and they assisted for attaining diagnosis after a showing a concern for a request for further investigations. 11 mothers (13.8%) were able to identify the baby’s diagnosis directly without any assistance, followed by 7 mothers (8.7%) who were told by a relative, and 3 mothers (3.7%) who were told of the problem by the doctor that their babies needed medical attention. 24 times mothers in the peripheral hospitals reported bad language like “I don’t have time to listen to you every day and every time.” 77 mothers in the periphery (90.6%) were not satisfied with the amount of time spent by the doctors in seeing their babies.

**Conclusion:**

Mothers of the neonates play great roles in identifying the illness of the newborn. Mother’s awareness of what might be needed during neonatal support strategies to improve neonatal care in both health facilities and the communities.

## Background

The neonatal period is only 16.7% of the first five years of life, but it contributes to 38-40% of deaths in children younger than 5 years (7.6 million to 10.5 million under-five deaths) [1,2]. The reduction in neonatal mortality rates globally accelerated between 2000 and 2010 (2.1% per year) compared with the 1990s, but was slower than the reduction in mortality of children aged 1-59 months (2.9% per year) [3]. Trends towards achieving MDG number 4 have shown that reduction of neonatal mortality rates ranged from 3.0% per year in developed countries to 1.5% per year in sub-Saharan Africa [2]. Social demographic patterns may contribute to impoverished mothers' level of involvement in neonatal health delivery and such factors have been associated with neonatal mortality [4,5].

Tanzania is among the top five countries with the highest rates of newborn deaths in sub-Saharan Africa [6,7] setbacks were highlighted as early as 2008 [8]. In northern Tanzania, maternal social demographic factors are explained to be the major cause of excess neonatal mortality [9,10]. However, in rural southern Tanzania, deaths at health facilities were higher (32.3 per 1000 live births) than those in the community (29.7 per 1000 live births) [11,12]. Performance of health care workers has become a main challenge in neonatal care [13] such that care at home after home delivery has proved to be less of a problem than care given at the health facility [14]. This leads us to believe that elements such as emotional support, parent empowerment, a welcoming environment with supportive unit policies, and parent education with an opportunity to practice new skills in neonatal care have an impact on improving neonatal care [15-17]. Mothers are the main stakeholders of neonatal care, and they should be involved in decision making. However, they rarely have been involved in neonatal care in the health facilities [18].

While significant research and massive international interventions have been put towards reducing maternal and neonatal mortality [7,19], very little has been done to include mothers’ perceptions and behaviours regarding childbirth as well as to determine the support mothers have received and wish to receive from hospital facilities [20,21]. If neonatal outcomes are to be improved, there is a need to focus on community members’ opinions, particularly the mothers, so as to foster appropriate hospital neonatal ICU care [22,23].

Health care is regarded as better when there is a good outcome and a higher patient satisfaction [24,25], which includes establishing a sense of trust, covering the patient’s need, and promoting dialogue and follow up of patients or sick neonates through parents [15,26,27]. These are important indicators for improving the quality of care [28]. However, in developing countries social-demographic factors of mothers, including area of residence, husbands’ age, maternal height, ethnicity, education, occupation, and cultural barriers have all been shown to affect neonatal health [9,29]. Additionally, there are some delays in receiving health care that influence mothers to have poor neonatal outcomes [30]. These delays include mothers’ failure to recognize problems, delaying a decision to seek care, and delaying to reach a health facility due to lack of transportation funding or distance [31].

Women's experience during childbirth has also been affected by the limited performance and unwelcoming behaviour of health workers [32] especially when there is a need for counselling and education [16]. Ultimately, mothers develop a reluctance to use maternity care, which therefore leads to poor neonatal outcomes [33]. Mothers in developing countries do not have options due to maternal education, husband's education, marital status, availability, cost, household income, women's employment, media exposure and having a history of obstetric complications, cultural beliefs and ideas about pregnancy [34]. It seems that mothers’ understanding towards responsibilities in postnatal care should be further studied to generate strategies [35] to improve neonatal care, such as those seen in west Africa at district level [36], which are lacking in Tanzania. There is evidence that there is poor record of care and lack of health workers motivation in northern Tanzania [37]. The affirmation of mothers’ opinions and suggestions in health care [38,39] can support a design for strategies to improve the quality of maternal and neonatal care, which will be key to helping Tanzania achieve the Millennium Development Goal Number 4 (MDG 4) of reducing child mortality [40-46]. Describing the expectations and experience of mothers during perinatal care will offer a road map to improving neonatal care. The aim of this study was to describe the quality of neonatal care in Kilimanjaro region based on mothers’ perspectives and experiences in neonatal care as a way to achieve MDG 4 for a northern Tanzanian context.

## Methods

The overall research design was a cross sectional study using quantitative and qualitative approaches. The study was conducted in the Kilimanjaro region located in north-eastern Tanzania. The study involved all 7 districts in the region where 13 peripheral hospitals and a tertiary referral hospital were purposively selected. Within each selected hospital, mothers of the admitted sick neonates were selected cross-sectionally and recruited in the clusters of the hospitals selected. All available eligible participants at the time of the hospital visit were approached for inclusion.

Data were collected from 13 peripheral facilities as well as at the zonal referral hospital from 26^th^ November, 2010 to 25^th^ April, 2011 112 guided semi structured interviews were done. Our questionnaire also included more open-ended questions so as to allow more insights from the mothers on issues that we did not ascertain prior to the interviews. Where necessary, notebooks were used for making notes on additional observations. We asked mothers about their social-economic and demographic background, and we then carefully enquired on their experiences regarding the health workers’ attitudes during reception, labour (if there was any), about explanations given regarding the neonate’s illness, attending the neonate, and follow up of the neonate. Where necessary clarifications were required, we used probes to get the innermost insights from the mothers. Mothers were asked about what could have been done better to improve the health care of their babies. When the answer was not specific, mothers would be probed for examples of incidences that justified what she had mentioned. We asked for what went well and what went wrong so as to assess patient satisfaction as described in the toolkit for high quality of neonatal care services [25].

To avoid a Hawthorne Effect, without prior notification, but with the ethical clearance and RMO letter, data was rapidly collected in one to two days for assessing the record of care in neonatal files and interviews with mothers at each facility we visited. Occasionally, three days were spent in a facility when additional hospital statistical data was required at the facility.

### Ethical consideration

The study was approved by the Kilimanjaro Christian Medical University Ethics Committee and a letter of introduction was also received from the Kilimanjaro Regional Medical Officer. Written consent and permission for hospital involvement was obtained from the senior medical officer of each health facility. Before conducting interviews and reviewing files of the neonates, written consents was obtained from the guardians (mothers) that provision of health care to their babies will be assessed and they will be interviewed in parallel.

### Data analysis

We analysed quantitative variables in STATA version 10 (STATA v10StataCorp, TX, USA). Chi-square was used to test the significance of difference between maternal sociodemographics, level of satisfaction with care, delays in care, and identification of illness between mothers enrolled in peripheral facilities versus those enrolled in the zonal referral hospital..

Qualitative narratives on discussions made by interviewers with mothers were written in narratives on open-ended space in the questionnaire and further additions in notebooks. Narratives were transcribed and coded and sub-coded into various categories with Excel 2007 so as to gather summary information. Qualitative information was summarized into different themes that were manually counted into main description of what could have been done better. We could generally group comments into categories of health worker performance, shortage of supplies, number of health workers, disturbances in health systems and a need for mothers' education and orientation. In addition to the above codes, we also recorded specific suggestions from mothers about what could have been done better.

## Results

We were able to assess mothers experience in neonatal care in 2 health facilities, 10 district hospitals, 1 regional hospital and 1 referral centre in the Kilimanjaro region. To simplify description, we henceforth refer to all facilities which refer seriously ill neonates to the zonal facility as peripheral hospitals and the zonal facility itself as the referral hospital. Within the peripheral facilities, there were a mix of missionary hospitals and public district hospitals. In the group of district hospitals, six were supported in infrastructure by missionary organisations and labelled as Designated District Hospitals (D.D.H.), and four of them were government based District Hospitals labelled as D.H.

There were 80 mothers of sick neonates interviewed in the periphery and 32 mothers in the referral facility. From the peripheral facilities, 59 mothers (73.7%) delivered at the facilities we visited, 11 mothers (13.7%) at other facilities, 7 mothers (8.8%) at home, and 3 mothers (3.8%) delivered on the way to the hospital facilities. At the referral centre we visited for reference, 23 mothers (71.8%) delivered at the facility where we found them, 8 mothers (25.0%) in another facility, and 1 mother at home (3.1%). Demographic distribution is shown in Table 1. There was no significant difference in social economic status between mothers attending peripheral facilities or the referral hospital (*χ*^2^(2) = 1.05, P value = 0.589). The difference in education levels between mothers who attended the peripheral facilities and the referral hospital was statistically significant (*χ*^2^(4) = 13.29, P value = 0.01).

**Table 1:** Demographic summary of mothers interviewed

**Age**	**Frequency (Proportion)**	**Frequency (Proportion)**
15-20	14 (17.5%)	11 (34.38%)
21-30	47 (58.75%)	16 (50%)
31-40	17 (21.25)	5 (15%)
41-50	2 (2.5%)	0 (0%)
**Education Level**	Frequency (Proportion)	Frequency (Proportion)
Never	1 (1.25%)	3 (9.38%)
Primary, incomplete	13 (16.25%)	0 (0%)
Primary, completed	42 (52.5%)	13 (40.68%)
Secondary School	19 (23.75%)	11 (34.38%)
Higher Learning	5 (6.25%)	5 (15.63%)
**Social-Economic Status**	Frequency (Proportion)	Frequency (Proportion)
Low Social-Economic status^†^	9 (11.25%)	2 (6.25%)
Moderate Social-Economic status^†^	70(87.5%)	29 (90.63%)
High Social-Economic status^†^	1 (1.25%)	1 (3.13%)

Key: ^†^ social economic status was graded by the use of roofing material, floor of the house, possession of radio or Television or both, Possession of Bicycle or Motorcycle or car.

For question whether mothers were able to identify their baby’s illness, 59 (73.8%) mothers in the peripheral hospitals reported that they noticed their babies had a medical problem after their request for further medical investigations. 11 mothers (13.8%) noticed the baby’s diagnosis themselves without any assistance, followed by 69 mothers (8.7%) Other 11 mothers (13.8%) noticed 248 the baby’s diagnosis themselves without any assistance, followed by 69 mothers (8.7%) who were told by a relative, and 29 mothers (3.7%) who were told of the problem by the doctor. At the referral hospital, there was a relatively higher proportion (65.5%) of women who noticed the baby’s problem by themselves. The difference observed between the groups was statistically significant (*χ*^2^(4) = 35.93, P value < 0.001).

**Figure 1:**
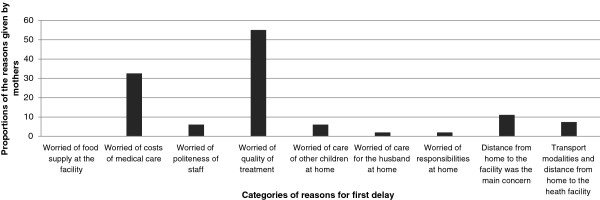
Reasons for delaying making decision to attend for medical services.

In the periphery facilities, 27 mothers (33.8%) reported to face problems in making a decision to seek care at a health facility, while at the referral hospital, 4 mothers (12.5%) reported to face problems. 49 responses were collected mothers who reported to face problems in making a decision to go to a facility (primary delay). The most common response was for quality of treatment at the facility reported 27 times (55.1%) followed by cost of medical care, reported 16 times (32.6%) Figure 1. Social/cultural issues were less frequently reported. Parameters for second delays could be captured as distance from home 5 times (11.1%) and combined distance and transport at a frequency of 3 times (7.4%) Distance was not found to affect mothers in attaining perinatal care at the first or tertiary level of referrals in the Kilimanjaro region. There is no statistical difference in the distance or location between the mothers attending peripheral facilities or the referral hospital (*χ*^2^(3) = 4.82, P value = 0.185).

For the delay on receiving neonatal care, 25 (31.3%) attempted to call a nurse or doctor for further check up of the baby. The average time taken was 13.8 minutes with a standard deviation of 11.4 minutes where the minimum time taken was 3 minutes and the maximum time was 45 minutes.

**Figure 2:**
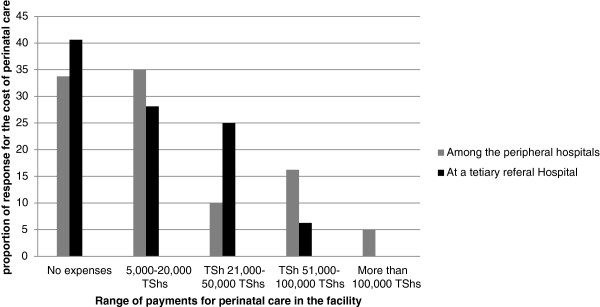
**Summary of cost of perinatal care as reported by mothers.** Note: 1 USD is equivalent to 1650 TShs by 1st March 2012.

One third of mothers in the peripheral hospitals reported having no expenses during care, however, this represents mothers who were not asked to pay for any services from admissions to the time we interviewed as well as those mothers who did not know how much they would be required to pay at the time of discharge. Patterns of costs are shown in Figure 2. The differences in expenses incurred by mothers between the two levels of care was not significant (*χ*^2^(4) = 7.53, P value = 0.110).

In terms of the levels of support and friendliness, there were 3 mothers at the periphery centres (3.7%) and 1 mother at the referral hospital (3.3%) who referred to the service provided as “not supportive at all”. “Partly supportive” was cited by 14 mothers at the periphery (17.5%) and 5 mothers at the referral hospital (16.6%). 27 mothers (33.8%) at periphery centres and 6 mothers at the referral hospital (18.7%) described the service as “mostly supportive”, and “very much supportive” was cited by 36 mothers (45.5%) at periphery centres and 18 mothers at the referral hospital (56.3%).

**Figure 3:**
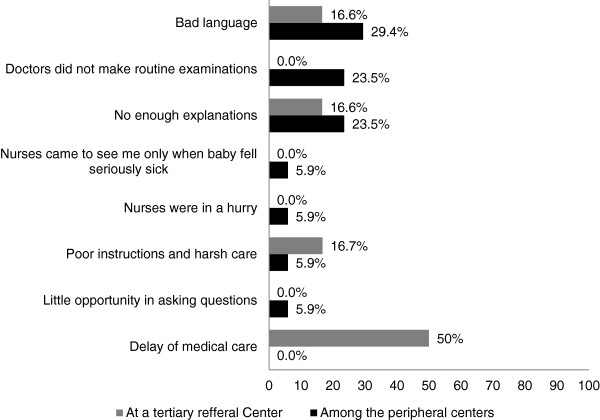
Mother’s complaints of unfriendliness of health workers.

Complaints of mothers regarding unfriendliness are shown in Figure 3, which demonstrates 24 instances where mothers in the peripheral hospitals reported bad language like “I don’t have time to listen to you everyday and every time” or “why did you came here if you know”. Mothers also complained that there was a lack of routine examinations by the doctors, which was cited by 20 mothers, and insufficient explanations on how to feed and offer a kangaroo method to a neonate was mentioned by 17 mothers. Other complaints included poor staff performance, such as harsh care. At the referral hospital, delay of medical care was a leading comment, being reported 15 times. Poor instruction and harsh care was reported 5 times, and mothers complained that the staff used bad language 5 times.

When asked about the amount of time spent with the doctors, 72 of the mothers (90.6%) were not satisfied with the amount of time spent by the doctors to see their baby. Combined, 38 of 80 mothers from periphery centres (47.5%) were in the category of too little or no opportunity to ask health workers questions about their babies. Mothers at the referral hospital reported much higher satisfaction, with 28 of them (87.5%) reporting that there was enough opportunity to ask questions. The differences of opportunities for mothers at two different levels of care (peripheral and referral) were statistically significant (Pearson *X*^2^(2) = 12.53 P value = 0.002).

**Figure 4:**
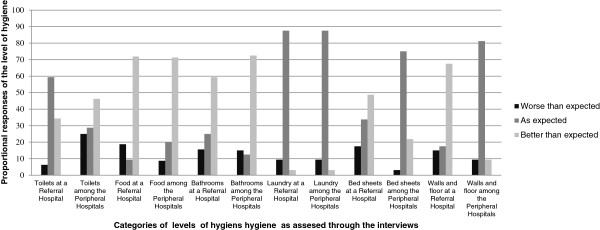
Summary of the levels of hygiene at the two levels of care.

By considering levels of hygiene (shown in Figure 4) by the cleanliness of the wall and floor, it is observed that in this category there are statistically significant differences between those at the peripheral and referral levels (*X*^2^(2) = 41.72, P value < 0.001).

### Qualitative results

A variety of responses were given by the mothers, however, the majority of the mothers in the peripheral hospitals (52.4%, n=105) showed a concern that there was need for improved performance and care given by the health workers. Among mothers who were disappointed in the health workers’ performance in the periphery, 76.4% (n=55) mentioned that they wished the health workers could provide more frequent visits to the baby or to be nearer to the baby. One mother, at F1 M_4_, aged 28 years, who was not told what the diagnosis was and could not remember the medication given to her baby commented, *“Nurses and doctors need to be close to mothers and babies; I think medical check up should be done every day.”* Other complaints included a lack of proper explanation to the mothers. At F6, M_46_, 36 years old mother, whose baby was floppy and coughing, explained that *“doctors and nurses need to be to be careful with their work all the time, because they keep giving me different information on the same illness of the baby.”* Other mothers were disappointed in the attitudes and politeness of the health workers. At F7 M_50_, 23 years old mother was told that her baby had febrile illnesses due to bacterial infection, and she mentioned that the nurses need to *“increase politeness to the mothers. They shouldn’t just simply explain to us what to do while not telling the causes of the illnesses.”*

**Figure 5:**
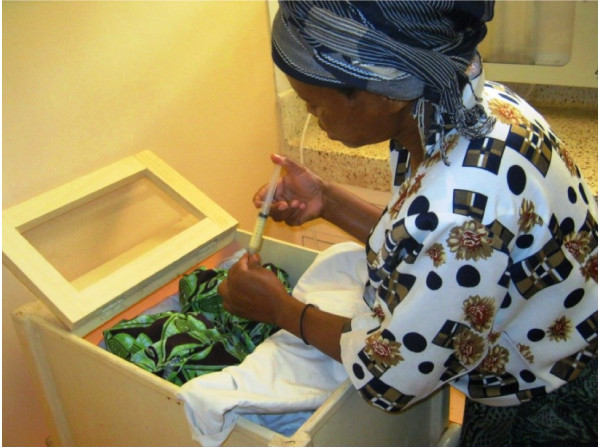
**The evidence of the impact of a mother after being well trained.** A well experinced mother of the neonate trying to feed a premature baby in the premature unit of the hospital facility in Kilimanjaro region. (consent obtained).

The proportion of mothers who recommended improved health worker performance from the referral hospital was lower than those from the periphery (16.2%, n=37). One mother, At F9 M_116_, 33 years old mother, whose baby was suffering from high fever and vomiting, commented, *“I think my baby was one of those who was not thoroughly checked and investigated. There is a need for an increase of investigations so as to identify the baby’s problem.”* This was noted by our observation that some neonates were kept in one incubator without a thorough investigation for the source of infection (Figure 5).

Among peripheral facilities, 14.3% of the mothers’ opinions mentioned a shortage of drug supply. At F3 M_31_, 25 years old mother had a baby with febrile illnesses due to a bacterial infection. She mentioned that “*Availability of medicine in the hospital should be improved.*” At F1 M_20_, 33 years old mother, whose baby had fever due to infection and presented with difficulty in sucking and bay was allocated on Ampicillin, Gentamycin and Cloxacillin. She explained, *“I wished there could be more drugs for the babies, because I felt disturbed to leave my baby to the neighbour while I move out for the pharmacy.”* At F1 M_5_, 23 23 years old mother, who had a baby with yellow coloration of the eyes and fever, was given a piece of paper with medication that she called injections given to her baby. She mentioned, “*We need more drugs for the baby, and I wish we were not supposed to buy at the pharmacy outside the hospital*.” There were no mothers interviewed at the referral hospital that mentioned drug supply to be a problem.

At the proportion of 13.3%, mothers gave opinions on a need for more staff. For example, At F10 M_70_, 19 years old mother brought her baby to the hospital because of febrile illness due to a bacterial infection and mentioned that her baby was given Christapen. She explained, “*the number of nurses should be increased so that they can better prioritise children’s health care.”* A shortage of staff is likely what can explain the aforementioned complaint of infrequent visits by the health workers.

Mothers interviewed in the peripheral facilities (13.3%) expressed concern over receiving little to no education from the health workers on how to properly care for their baby or about the illness of the baby. At F1 M_9_, 20 years old mother was not told the diagnosis or what medications were given to her baby. This mother commented that *“they did not explain my baby's problem, and I was not taught how to take care of my baby at home.”* F14 M_102_, 27 years old mother, who had a baby with high pitch cry and bleeding of the umbilical cord, had a similar comment. She said*, “staff should inform mothers about the illness of their babies and what the outcome should be after giving the baby medicine.”* At F8 M_60_, 28 years old mother commented on how the attitudes of the health workers affected her. She wished the workers had *“educated us on what our mistakes were instead of just becoming furious with us.”*

Among the peripheral facilities, 4.8% of the mothers’ opinions involved an issue of facility shortages, including a shortage of equipment, space/wards or beds. At F9 M_65_, 34 years old mother explained that “*increasing the number of beds in the ward here is very important, because we were two in one bed before delivery.”* While facility shortages did not seem to be a major concern among mothers at the peripheral facilities, many mothers at the referral hospital seemed to notice facility problems. 45.9% of the opinions of mothers interviewed at the referral hospital mentioned facility shortages to be their major concern. At F14 M_96_, 22 years old mother described her room as very hot and said that she wishes “*to see an increase in the number of wards, rooms and beds in this facility.”*

A small proportion of mothers (2%) discussed issues in hygiene among the peripheral facilities. One mother, At F7 M_52_, 20 years old mother, mentioned that the facility should “*increase the level of hygiene here*.”

## Discussions

Mothers in Kilimanjaro region are well informed on what need to be done in the health facilities. This report adds on the comments from Uganda that shows community interventions play a great role reduction of neonatal deaths [47].

Higher awareness among mothers of Kilimanjaro explains the low proportions of first delay in the region and assist mothers to identify a baby who need immediate care. Appraisal of care through mothers report a persistence of bad language and limited instructions to mothers have remained to be a problem in Kilimanjaro region, as have been described in studies for older children [48,49]. Mothers are the good source of best practice by health workers on knowledge, attitudes as reported from studies of China, in Asia [50]. Our study emphasises on detection of illnesses from Mothers as source. Further, when sufficiently educated, mothers can by ask health professionals for further check up of their babies and hence support triage systems [51].

Kilimanjaro region experienced Kilimanjaro region covers a relatively small area, a first delay (making a decision to go to a facility) more frequently than a second delay (transportation). These finding are relatively similar to studies western Tanzania [31] but not in proportional differences. Half of the hospitals in Kilimanjaro region are church based and well equipped [37]. Kilimanjaro region has the potential to serve as a role model region for quality of neonatal care during the count down of MDG 4 for Tanzania.

Although we did not explore the proportion of mothers who had health insurance, generally, mothers who did not report to pay anything at the time of interview were worried that there would be some costs incurred at the time of discharge. Majority of the mothers had already paid 5,000 TSh (US$3) to 20,000 TSh (US$12) for neonatal care at the time of interview. This is not in line with household income, which is only 40,000 TSh (US$24) on average, and the per capita monthly income is 12,667 TSh (US$7.60) [52]. The cost of neonatal care remains to be compromising the family financial stability and provision of care at the facilities.

## Conclusions

Despite their lower educational and social economic status, mothers have strong impact on detection of illness of their babies after birth and the outcome of subsequent clinical care.

### Recommendations

There is a need to introduce special medical training programme for mothers on neonatal care training in the primary health care facilities that focuses on community intervention in which mothers should be well involved.

## Abbreviations

ICU: Intensive care Unit; F1: First facility to be visited; F2: Second facility visited; F3: Third facility visited; M1: First mother to be interviewed; M2: Second mother to be interviewed; M3: Third mother to be interviewed; MDG: Millennium Development Goal; RMO: Regional Medical Officer; STATA: A complete, integrated statistics package of statistical software used for data analysis.

## Competing interests

The study was funded by the Ministry of Health and Social Welfare as the first authors’ prerequisite study for the completion of MSc Clinical Research at Kilimanjaro Christian Medical University. Additional working support and expertise was given by volunteers who were medical personnel and students from developed countries. There has been no competing interest for funding of the study. There have been no reimbursements, fees, funding, or salary from any organization that might be affected anyhow by this publication, neither now nor in the future. The author does not hold any stocks or shares in an organization that may in any way might be affected by this publication. The authors are currently applying for the intervention phase of Quality of neonatal heath care in Kilimanjaro region. There have been no experiences of non-financial competing interests in any form of political, personal, religious, ideological, academic or intellectual.

## Authors’ contribution

BM developed a concept of research work, proposal development, data collection, database development, analysis, report writing and writing of the manuscript. ER was the mentor and advisor of the designs of research in pediatric care. She also supported scientific writing of the report and the manuscript. RM was the official local supervisor and a consultant of qualitative techniques from community health department at Kilimanjaro Christian Medical University. She supported the work of report writing and manuscript development. NLI performed data analysis in the qualitative narratives. SAPW supported database development and initial data collection. JAM and FAM worked in data collection in the hospitals and data entry. All authors read and approved the manuscript before publishing.

## Author’s information

BM is the Tanzanian medical doctor and clinical researcher at Kilimanjaro Clinical Research Institute of Kilimanjaro Christian Medical Centre, Moshi Tanzania. RM, the supervisor of the principal investigator is the head of community health department at Kilimanjaro Christian Medical University. NLI, a public health specialist from United States supported qualitative analysis of the research work. She graduated from Seattle Pacific University with a Bachelors of Arts in Political Science/International Affairs. she is conducting health research studies and providing basic health care among Tanzanian street children. ER is a Paediatrician, internist and researcher who direct collaborative research programs between Duke University and Kilimanjaro Christian Medical Centre. SAPW is a language specialist from United States and a medical student at Tufts University School of Medicine supported database development. She volunteered for children health programs in Moshi in 2010 and 2011 before joining medical school. JAM is a medical student from University of Georgia, Athens, US. FAM is a health volunteer at Uru Mawela Parish, worked on the data collection and data entry for Swahili version of mother’s interview.

## Pre-publication history

The pre-publication history for this paper can be accessed here:

http://www.biomedcentral.com/1471-2431/13/68/prepub
